# Bridging the gap between DeafBlind minds: interactional and social foundations of intention attribution in the Seattle DeafBlind community

**DOI:** 10.3389/fpsyg.2015.01497

**Published:** 2015-10-09

**Authors:** Terra Edwards

**Affiliations:** Department of Linguistics, Gallaudet UniversityWashington, DC, USA

**Keywords:** intention attribution, deictic reference, pointing, DeafBlind, Tactile American Sign Language, deictic integration, practice

## Abstract

This article is concerned with social and interactional processes that simplify pragmatic acts of intention attribution. The empirical focus is a series of interactions among DeafBlind people in Seattle, Washington, where pointing signs are used to individuate objects of reference in the immediate environment. Most members of this community are born deaf and slowly become blind. They come to Seattle using Visual American Sign Language, which has emerged and developed in a field organized around visual modes of access. As vision deteriorates, however, links between deictic signs (such as pointing) and the present, remembered, or imagined environment erode in idiosyncratic ways across the community of language-users, and as a result, it becomes increasingly difficult for participants to converge on objects of reference. In the past, DeafBlind people addressed this problem by relying on sighted interpreters. Under the influence of the recent “pro-tactile” movement, they have turned instead to one another to find new solutions to these referential problems. Drawing on analyses of 120 h of videorecorded interaction and language-use, detailed fieldnotes collected during 12 months of sustained anthropological fieldwork, and more than 15 years of involvement in this community in a range of capacities, I argue that DeafBlind people are generating new and reciprocal modes of access to their environment, and this process is aligning language with context in novel ways. I discuss two mechanisms that can account for this process: *embedding in the social field* and *deictic integration*. I argue that together, these social and interactional processes yield a deictic system set to retrieve a restricted range of values from the extra-linguistic context, thereby attenuating the cognitive demands of intention attribution and narrowing the gap between DeafBlind minds.

## Introduction

This article analyzes some of the social and interactional mechanisms that constrain pragmatic acts of intention attribution among DeafBlind people in Seattle, Washington. In particular, it focuses on the use of pointing signs and the means by which potential referents in the environment are narrowed down. In visual signed languages, pointing signs can be used gesturally, but they are also recruited by the grammar, taking on a range of linguistic functions (Friedman, 1975; Klima and Bellugi, 1979; Supalla, 1982; Petitto, 1987; Padden, 1988; Engberg-Pedersen, 1993; Liddell, 1995; Taub, 2001; McBurney, 2002; Meier, 2002; Rathmann and Mathur, 2002; Pfau and Steinbach, 2006; Pizzuto, 2007; Coppola and Senghas, 2010; Meier and Lillo-Martin, 2013; Gökgöz et al., 2015). Evidence for this includes, among other things, that some pointing signs are acquired by children according to developmental patterns similar to learners of corresponding spoken language forms (Petitto, 1987; Pizzuto, 2007: p. 292; Gökgöz et al., 2015), they appear to have syntactic distributions that are the same as corresponding elements in spoken languages (Padden, 1988), but different from co-speech pointing gestures (Cormier, 2014:4, *Cf*. Johnston, 2013), and they are subject to visual and processing constraints that apply to linguistic, but not gestural phenomena (Siple, 1978; Emmorey, 2002).

There are, however, unresolved theoretical issues regarding characteristics of pointing signs that are difficult to account for from phonological, morphological, and syntactic perspectives (Mathur, 2002; Pizzuto, 2007). These problems have been approached from many different angles (see Mathur and Rathmann, 2012, for a review), and yet, scholars are converging on the fact that pointing signs, no matter how far into the grammar they penetrate, cannot be adequately described via linguistic analytics alone (Liddell, 2003; Dudis, 2004; Johnston, 2013; Meier and Lillo-Martin, 2013; Cormier, 2014). This pushes pointing in signed languages into the realm of pragmatics, where questions of intention attribution inevitably arise (Grice, 1971; Levinson, 1983; Searle, 1983).

In the sign language linguistics literature, intention attribution, and more generally, speech act theory, has played a fairly limited role in addressing problems associated with pointing. Instead, concepts such as cognitive capacity, gesture, and iconicity have been central and from those perspectives, constructs such as “real space” (Liddell, 2003: p. 82), “gestural space” (Rathmann and Mathur, 2002: p. 144), and “iconic prototypes” (Sandler, 2012) have been proposed. These constructs tend to assume a non-problematic relationship between representations (both linguistic and cognitive) and embodied experience. Liddell for example, defines real space as “a person's current conceptualization of the immediate environment based on sensory input” (Liddell, 2003: p. 82). Real space is isomorphic with the conceptualizer's experience of the environment, and is assumed to be reciprocal across the group of language-users since “[i]n general, real space lines up well with physical things in the world” (Liddell, 2003: p. 84). From this perspective, intention attribution seems pretty simple—you and I inhabit the same world and/or representation of it, so when I point to an object or location in real or imaged space, the object of my attention will likely be self-evident to you^1^.

For DeafBlind people in Seattle, however, seamlessness between experience and representation can rarely be assumed. Most members of the community are born deaf and become blind slowly. Everyone becomes blind in different ways and at different rates. Therefore, sensory capacities and habitual modes of sensory orientation vary significantly across the group. These differences are compounded by differences in race, ethnicity, gender, age, disability, socioeconomic status, sexual orientation, and school experience (i.e., growing up in a residential school for the deaf vs. a deaf program within a hearing school, etc.). Furthermore, tactile reception of Visual American Sign Language (VASL) was, until recently, the only available choice. Since VASL emerged and developed among sighted people, and is therefore built around visual modes of access and orientation, it is only partially perceptible via tactile reception, much as spoken English is only partially perceptible via lip-reading (see Edwards, 2014b). In other words, for DeafBlind people, the systems of representation historically available to them are shaped by a world that they can no longer access. In addition, authority accrued to sighted social roles, and legitimacy accrued to visual modes of communication, therefore, in order to maintain one's position and status in the social order, tactility had to be avoided. In the past, these barriers were considered too great to surmount and direct communication between DeafBlind was rarely attempted (Edwards, 2014a: pp. 86–90). Instead, DeafBlind people communicated via sighted interpreters. However, since 2007, a socio-political movement known as the pro-tactile movement has opened up new possibilities for direct communication between DeafBlind people.

The pro-tactile movement is based on the idea that all human activity can be realized via tactile-kinesthetic channels, including interaction and language-use. Therefore, interpreters are not necessary for DeafBlind people to interact with each other or their environment. However, in order to legitimize practices built around tactile modes of access, social restrictions on touch have to be relaxed and experimentation encouraged. In 2010 and 2011, a series of 20 pro-tactile workshops was led by two DeafBlind instructors for 11 DeafBlind participants with these aims in mind. This paper focuses on several interactions between DeafBlind people that took place as part of the pro-tactile workshops^2^. The interactions were videorecorded by a team of three videographers from multiple angles (120 h of video data was collected in total). Sequences of communicative activity where DeafBlind people coordinated and directed each other's attention to particular dimensions of setting were subsequently isolated, and the ways in which pointing signs were produced and responded to were considered^3^. Examining these moments among DeafBlind people in Seattle offers unique insight into how language and context are brought into alignment with our embodied experience of the world.

In Speech Acts and Intentional States from a Practice Perspective, I begin with a discussion of intention attribution viewed through the lens of practice theory (Giddens, 1979; Bourdieu, 1971, 1990 [1980]; Hanks, 1996, 2005a,b; Edwards, 2012, 2014a,b). From this perspective, embodied knowledge takes on a crucial role for language-users as they work to converge on specific, pragmatically situated meanings and effects in interaction. I argue that these embodied forms of knowledge arise in dynamic tension with structured and historically pre-given fields of social and interactional activity. In Embedding in the Social Field, I consider the effects of these tensions on language-use. Drawing on Hanks (2005b), I argue that embedding in the social field involves the legitimation of new styles, modalities, and genres, as well as the authorization of some language users (and not others) to evaluate linguistic forms and communicative practices as correct, appropriate, polite, or not. I argue that this dual constraint of legitimation and authorization restricts the range of feasible moves and interpretations in interaction among DeafBlind people in ways that simplify the cognitive tasks required for intention attribution. In The Deictic System and the Deictic Field, I ask how these two constructs work in tandem to structure deictic reference (Bühler, 2001 [1934]; Hanks, 2005a). When a deictic sign is instantiated, contextual values must be retrieved and coordinated, and patterns in retrieval have an effect on the internal organization of the language. I call this process “deictic integration.” Via detailed analysis of interactional sequences among DeafBlind people, as well as attention to their metapragmatic commentary, I show how deictic integration is accomplished in the workshops. In Deictic Integration and Appropriate Pointing in TASL: Embedding and Integration in the Social and Deictic Fields, I argue that in conjunction with embedding in the social field, deictic integration is giving rise to a deictic system in Tactile American Sign Language (TASL), which diverges from the visual system on which it is scaffolded. Evidence for this claim includes an emerging distinction between demonstratives and locatives in TASL represented by a difference in movement (tapping vs. tracing, respectively). I show how these changes emerged as certain practices for pointing were deemed appropriate and others were deemed inappropriate by DeafBlind leaders, who are invested with the requisite authority. I conclude in the final section, with some reflections on the role of deictic integration and embedding in the social field for simplifying the task of intention attribution from the perspective of the DeafBlind participant. In particular, I emphasize the importance of socially transmitted forms of embodied knowledge in fitting the linguistic system to particular fields of activity, thereby narrowing the gap between DeafBlind minds.

## Speech acts and intentional states from a practice perspective

When people apply linguistic resources in the speech situation, they are not only producing semantic meanings; they are also performing pragmatic actions such as informing, requesting, and asserting (Austin, 1965; Searle, 1969; Grice, 1989). And yet, when utterances are taken out of context, the pragmatic layer can collapse, revealing a kind of “residual semanticity” or indeterminacy that can be manipulated by speakers to deny specific inferences: “Thus, the characteristic speaker's denial of speech offensive to the hearer takes the form of ‘all I said was…’” (Silverstein, 1976: p. 47). Reducing an utterance in this way produces many possible interpretations, which must be narrowed to generate specific meanings and effects in interaction. One of the ways that participants accomplish this is by attributing communicative intentions to their interlocutor (Grice, 1971; Levinson, 1983; Searle, 1983).

Intention, in the sense of meaning to do something, is just one of many intentional states. Broadly construed, a mental state is intentional insofar as it is directed toward an object or state of affairs (Searle, 1983: pp. 1–37). Other intentional states include, for example, belief, love, elation, anxiety, irritation, and remorse^4^ (Searle, 1983: p. 4). It is in this broader sense that the term is taken up here. Intentional states correspond in many ways to speech acts; speakers can insist that their interlocutor leave the room in much the same way as they can believe, fear, or hope their interlocutor will leave the room (Searle, 1983: pp. 5–6). These kinds of correspondences come together in Searle's “conditions of satisfaction,” including, for example, his sincerity condition^5^. Each time an illocutionary act is performed, an intentional state is expressed via the same propositional (or representative) content (Searle, 1983: p. 9). Insofar as the intentional state and the illocution correspond, the speaker satisfies the sincerity condition. For example, if I say, “It is snowing,” I have produced an assertion (speech act), which corresponds to the belief (intentional state) that it is snowing. If I believe it is snowing when I assert that it is snowing, I have satisfied the sincerity condition. The sincerity condition is one among many, which link utterances (and other representative content) to a psychological and/or illocutionary mode, thereby specifying its meaning or effect.

However, anthropologists have shown that such conditions are culturally and historically specific, that they presuppose certain notions of personhood, and they can be more or less attenuated in different communicative contexts (e.g., Silverstein, 1976; Rosaldo, 1982; Duranti, 1984; Ochs, 1984; DuBois, 1987; Hanks, 1990; Kockelman, 2010). My aim is to build on this work by considering the role of embodied knowledge and practical circumstance in structuring the convergence of the speaker and addressee's intentional states on objects of reference in the immediate environment (Bourdieu, 1971, 1990 [1980]; Giddens, 1979; Hanks, 1996; Edwards, 2012, 2014b; Hanks, 2005a,b). To this end, I begin with the practical communicator, who exists in a world of routine, where much of what is said is anticipated and much of what is done could be done without saying much (Hanks, 1996). Informed by practice theory, I assume that patterns that emerge out of that regularity do not inhere solely in the linguistic system, nor can they be isolated in a static and detachable set of conditions or rules. Rather they cohere in the relations between the language-user, the language, and the specific fields of socio-historical and interactional activity where each is shaped. Embedded in routine patterns of embodied activity, the cognitive tasks required for generating pragmatically situated meanings appear less demanding than they might otherwise appear. In what follows, I outline three key concepts required for understanding intention attribution in a practice framework: habitus, field, and embedding.

### Habitus

Habitus is an acquired system of generative schemes, which predisposes actors to perceive, think and act in ways that feel correct, appropriate, and polite (Bourdieu, 1990 [1980]: pp. 52–65). Individuals share a habitus insofar as they are subject to social and material conditions that reinforce a ground of common sense ideas and behaviors, which, in turn, tend to reproduce the conditions that gave rise to those ideas (Bourdieu, 1971: p. 80). This circular process tends to convert history into second nature and in doing so, harmonizes the practices of the group in ways that are not transparent to its members. Harmonization is most apparent, analytically, in non-reflective patterns of thought, action, perception, and navigation^6^. Individual differences can only be evaluated reciprocally against the backdrop of a common habitus (Bourdieu, 1971: pp. 81–86).

Frames for evaluation, which tend to restrict possibilities for action, are an integral part of the habitus. These frames derive from the Aristotelian notion of *hexis*: an intention (or desire) to act together with reflexive judgments of that intention, guided by, or weighed against, frames of social value and meaning (Hanks, 2005a: pp. 69–72). Under the influence of Merleau-Ponty, Bourdieu's notion of hexis shifts analytic attention from the mind to the habituated activity of the body:

The evaluative perspective, once embodied, emerges as active perception, and the intentional states of desire and purpose become the inclination of body posture (Hanks, 2005b: pp. 71–72).

While Bourdieu locates hexis in the body and its dispositional tendencies, Giddens locates this kind of reflexive monitoring on three, distinct planes of consciousness: practical, discursive, and unconscious (1987: pp. 1–49). I focus here on the first two: practical and discursive consciousness. Practical consciousness accounts for “the tacit knowledge that is skillfully applied in the enactment of courses of conduct, but which the actor is not able to formulate discursively” (Giddens, 1979: p. 57). While practical consciousness accounts for what actors know how to do, discursive consciousness accounts for the knowledge actors are able to talk about.

Among DeafBlind people, dimensions of practice that would normally remain tacit are projected onto a discursive plane. This provides an unusual opportunity to see how embodied modes of knowledge contribute to the narrowing of interactional potentials in practice. For example, during the pro-tactile workshops, a group of DeafBlind people were playing a tactile version of charades. Someone would enact a character or person, everyone would explore the enactment tactually, and then they would take turns guessing who it was. After one of these games, an instructor asked a participant about his experience^7^.

Instructor: Was the game over there fun?

Participant: Yeah, but Chantelle had us all stand up while she did an elaborate performance of Marilyn Monroe. Then when we went to sit down, we all ran our heads into each other.

Instructor: Maybe if you did this [instructor puts hand on participant's shoulder, signs ready with the other hand, and begins to sit down], maybe that would work. Coming up with things like that, that's called “pro-tactile.” We have to be creative, because we can't see. We need new ways of coordinating group actions, like sitting down [they experiment with sitting down while touching each other's shoulders]. Maybe like that? [both smiling]. Do you think it's a good idea? [Participant: yes]. Great! I'll tell Adrijana about it, OK? [Participant: yep].

Here, DeafBlind participants are talking about a breakdown in practical sequence, where an embodied disposition that works well for the sighted leads to injury. When breakdowns like this occur, practical activity becomes an object of discursive reflection as the instructor and the student explicitly talk about, and try out, different combinations of communicative cues and body postures. After a few attempts, they agree on a particular strategy and the instructor says she will tell her co-instructor (Adrijana) what they have come up with. This shows that a strategy has been chosen and legitimized, making it a candidate for the communicative repertoires collaboratively constructed in the workshops. This process gives rise to novel practices while also linking them to social, evaluative frames associated with correctness, politeness, and appropriateness. Innovations that stick recede from discursive consciousness into practical consciousness. Part of what determines whether something will stick, and therefore recede, is the degree to which the practice is commensurate with the emergent, reciprocal body-schema of pro-tactile people.

### Reciprocity and the body schema

In a practice framework, the body schema is neither a representation of the body, nor a mere physical fact about the body (Hanks, 2005a: p. 69). Rather, it accounts for the “momentary grasp that actors have of being a body” (Hanks, 2005a). When the Marilyn Monroe charade was over and the participants began to sit down, their heads collided because their grasp of being a body, or their body schema was not commensurate with tactile modes of access. The collision presented an opportunity to bring representation, practice, and the physical surround into alignment. Practical strategies that do not lend themselves to such alignments tend to fall away over time as an inhabitable world coheres.

In a coherent, inhabitable world, hexis and the body schema work together to generate a reciprocity of perspectives (Schutz, 1970: p. 183). Where there is reciprocity, shared evaluative frames are applied to the reflexive grasp that DeafBlind actors have of being a body. Without this kind of reciprocity, people collide and are injured. They also have difficulty communicating, and these two facts are not unrelated. Where perspectives on the physical and social world are not reciprocal, propositional content appears under no particular perspective; the pragmatic layer never quite crystalizes, and the indeterminacy of language becomes a persistent, practical problem. The body plays a crucial role in addressing such problems, not as a physical or representational mechanism, but as the site of a reflexive grasp that social actors have of being a body. However, the tactile body demands relations to the world and to other people, which may appear inadmissible from a social perspective. In order to account for these constraints and their reconfiguration among DeafBlind people in Seattle, I appeal to the notion of the *social field*.

### The social field

The social field is a structured space of positions and roles, along with the historically specific means by which those positions and roles are occupied by social actors (Hanks, 2005a: p. 72). In the social field, speaking is a means of position-taking, which is dually constrained by legitimation and authorization (Hanks, 2005a: 72–73). Legitimation accrues to styles and genres of language use, knowledge of which is limited by social and economic position. Limitations on who has access to legitimate styles and genres restrict access to power, reinforcing unequal power relations. Authorization, on the other hand, is invested in the actors themselves, via the social roles they occupy (Hanks, 2005a: p. 76).

For DeafBlind language-users, dynamics in the social field include not only genres and styles of language-use, but also the relative legitimacy of different channels through which linguistic signs are exchanged (i.e., visual-kinesthetic vs. tactile-kinesthetic), the modes of access used to link linguistic signs to people, things, and events in the environment (i.e., memory, perceptual access, shared knowledge), and the relative authorization of social actors who habitually draw on and reproduce those channels in reciprocal ways (i.e., “visual people” vs. “tactile people”). Historically, visual channels and modes of access accrued more legitimacy than their tactile counterparts (Edwards, 2014b). Therefore, as DeafBlind people competed for resources in the social field, it was advantageous for them to continue communicating via visual channels and modes of access, even after they had become blind^8^. This meant that they did not have direct access to things like body posture, eye-gaze, and other embodied behaviors, which are transmitted by the habitus. Therefore, there was no way for one DeafBlind person to evaluate another against shared frames of social value. Instead, they relied on sighted people to share their interpretations and impressions. DeafBlind people were always removed from the embodied knowledge required for position-taking in the social field.

The inception of the pro-tactile movement brought with it a reconfiguration of social roles and positions, new ways of linking evaluative frames to embodied experience, and novel patterns in position-taking. Rather than accruing legitimacy by communicating as sighted people do, an internal hierarchy was established *within* the Seattle DeafBlind community. A small minority of DeafBlind signers, who were, importantly “tactile people” emerged as leaders and they applied their authority in judgments about the correctness of certain linguistic forms and communication practices. As a result, some embodied behaviors (and not others) became legitimate ways of being smart, polite, interesting, “culturally DeafBlind” and so-on. Patterns in language-use were caught up in this broader transformation, and novel linguistic forms began to mark new social distinctions (Edwards, 2014b). From there, pro-tactile practices could be used to acquire resources in the social field (e.g., prestige, membership, employment, etc.), without relying on the impressions, opinions, or interpretations of the sighted.

For example, in the following exchange, Lee, one of the instructors, identifies some linguistic forms and practices as appropriate, and others as inappropriate. In doing so, she is also legitimizing tactile modes of access to the environment and downplaying the necessity of visual access^9^.

Lee: The announcements for today are about the new rules. First, the video people—[…]—you're not allowed to talk with them. You're not allowed to ask them, as sighted people, where things are or where people are. So the film people are “not here.” That's crucial. […] That's the first rule. […] The second rule is that you have to be assertive—and feel around! You can't just stand there and wait for someone to tell you what to do.

Rules like this pushed participants toward tactile modes of access and exchange. DeafBlind leaders naturalized the practices that emerged as a result by labeling them culturally appropriate, correct, polite, and “pro-tactile.” For example, Adrijana explained to one of her students that

if someone is eating, and you touch their arms and their face, you will figure out they are eating. Then you know to leave them to their meal. It's the same thing with any other activity someone might be doing. You feel their arms, and then that leads you to some other part of their body, maybe their hands, and then you know what they're doing and how to interact with them. The point is that touching people for the purpose of gathering information is perfectly acceptable. So that is, in essence, what today's class has been about. […]

Adrijana is inviting her student to reconsider habitual, dispositional ways of interacting with others and with the environment. The appropriateness or inappropriateness of these practices is deeply ingrained from childhood, so abandoning established practices feels like a risk. Because Adrijana is invested with the requisite authority, her students took her up on her invitations, regardless. Where they felt resistance, they were encouraged to reflect. In one workshop, a participant identified the relative physicality of tactility and visuality as a potential problem for the pro-tactile movement. She argued that sighted people don't understand the range of things that touch can do, and too often assign sexual or romantic meanings to tactile signals.

They have to understand that touching is about feeling—its about having access to emotion—just like they have through vision. Touch is no more physical than vision.

These kinds of social facts—for example the commonly held idea that touch is more physical than vision—become apparent to DeafBlind people when they try to substitute tactile communication strategies for visual ones, and have the reflexive sense that they are doing something inappropriate. In response, Adrijana insisted that DeafBlind people must not comply with those impulses. Instead, she encouraged them to apply pressure to established social norms (i.e., join the pro-tactile social movement), or else suffer the effects of isolation:^10^

When people use their eyes for seeing, that causes them to feel. When people use their ears for hearing, that causes them to feel. [M]ost DeafBlind people have been missing out on feeling because we've been so focused on [language], and that's all. But there's this whole environment around us—a whole world—and we can't feel it. So that's why [the pro-tactile movement] is so important and why it has to include ways for us to feel things again. […] We need more stimuli for our bodies to interpret. All of that is part of “pro-tactile.”

Adrijana is not arguing that DeafBlind people are physically incapable of “feeling the world.” Rather, she is arguing that tactile modes of knowing have historically been limited by excessive social restrictions. Relaxing those restrictions requires new relations to be established between the habitus, the linguistic system, and the social field where touching is evaluated. I analyze this process as a kind of *embedding in the social field* (Hanks, 2005b).

## Embedding in the social field

Broadly speaking, embedding is a process through which highly schematic form-meaning correspondences undergo reshaping, conversion and transformation as contextual values are retrieved (Edwards, 2014b; Hanks, 2005b: p. 194). Through patterns in retrieval, the linguistic system is aligned with its contexts of its use, generating a restricted range of feasible interpretations. Four mechanisms of embedding have been proposed: practical equivalences, counterparts, rules of thumb (Hanks, 2005b) and integration (Edwards, 2012: pp. 52–63, 2014a: pp. 26–27). Practical equivalences, counterparts, and rules of thumb transform the meaning associated with forms as they are instantiated. Integration, in contrast, affects both form and meaning.

Embedding in the social field involves: (1) the legitimation of certain styles, modalities, and genres of language-use for taking up recognizable social positions, along with the embodied knowledge necessary to do so, and (2) authorization of some language-users to evaluate linguistic forms and communicative practices as correct, appropriate, polite, or not. In the social field, the effect of an utterance will be different depending on who produces it and what social position that person occupies. For example, in Yucatec Maya, an utterance produced by a shaman about a divining crystal will have a particular effect because of the authority invested in him and the social position he occupies, just as a radiologist's position authorizes him to interpret x-rays (Hanks, 2005b: p. 202). However, position is not enough. Legitimate modes of language-use, body posture, dress, overall comportment, and other aspects of practice must be convincingly enacted as well.

Legitimation and authorization constrain position-taking, thereby restricting the range of feasible moves in any interaction and the feasible interpretations of any utterance. In a practice framework, these restrictions are not listed a priori as maxims (Grice, 1989) or conditions (Austin, 1965; Searle, 1983). They are instead historically specific relations that cohere between: (1) actors, (2) social roles and positions, along with the structures they fit into, and (3) the embodied and linguistic knowledge required for taking up those roles and positions in legitimate ways. These relations are amenable to ethnographic, historical, and interactional analysis, and they have a role in shaping the internal organization of the language.

However, position-taking in the social field does not have a direct or determinate effect on the linguistic system. Rather, the social configuration of the body acts indirectly on the language as the ground against which reference is achieved. In other words, in order to individuate an object of reference in the immediate environment, the language must be aligned with the capacities of the body, the physicality of the world, and the reciprocal modes of access that are established across a group of language-users. In order to grasp these dimensions of practice, a shift in analytic perspective from the social to the deictic field is required.

## The deictic system and the deictic field

The deictic system and its corresponding deictic field structure how people refer to objects and events in the immediate environment (Bühler, 2001 [1934]; Hanks, 2005a). The deictic system is composed of semantic elements which are organized by contrastive opposition (e.g., *this* is not *that*). These oppositions contribute to the definiteness of reference, or the capacity of speaker and addressee to pick out a bounded thing among other things. Deictic signs also direct the attention of the addressee to the object by way of mutually accessible relations; this is the directivity of reference. While definiteness derives from the deictic system, directivity derives from the deictic field, where patterns in memory, sensory perception, navigation, and modes of attention, cohere to generate pathways, channels, grids, and coordinate schemes that speaker and addressee draw on to converge on an object. Therefore, all deictic signs are composite, composed of both “symbols” and “signals” (Bühler, 2001 [1934]: p. 99). Any time a deictic sign is instantiated, values must be retrieved from two distinct sources: the deictic system and the deictic field.

Given stable and reciprocal sensory capacities, relations of embedding between the two should be so seamless that reference to objects in the immediate environment feels self-evident, concrete, and natural to the language-user (Hanks, 1990: p. 5). However, in the context of radical shifts in sensory capacity, this apparent concreteness is disrupted, and the means by which the deictic system and the deictic field are brought into alignment is revealed.

The deictic system also registers social relations in an indirect way by aligning the grammar with modes of access that are reciprocal across a group of language-users (Edwards, 2014b). Modes of access include patterns in how the body perceives, moves through, remembers, and inhabits its environment. Any time signer and addressee converge on a referent in the immediate environment, modes of access in the deictic field must be coordinated. Analytically, the body must be viewed under distinct perspectives in the social and deictic fields^11^. However, in practice, the body that grounds reference is also the body that takes up positions in the social field. If a group of DeafBlind language-users has been socialized to avoid touching objects in their environment, it will be difficult to converge on an object of reference that is available via strictly tactile modes of access.

Therefore, social and deictic pressures are dually exerted on the linguistic system via the body. Nevertheless, as mentioned before, distinct analytic approaches are required for grasping the social and deictic processes that exert those pressures. In the social field, the analyst aims to understand how particular styles, genres, and channels are differentiated and legitimized for purposes of position-taking. In the deictic field, the analyst focuses instead on how pathways, relations, and dynamics in the environment are made reciprocal across a group of language-users as signer and addressee converge on objects of reference. Possibilities for how pathways in the deictic field can be organized are constrained at the outset, since many of the routes, relations, and modalities that could link speaker and addressee to the object given the physical and cognitive capacities of humans, are ruled out on social grounds in historically contingent ways.

## Deictic integration

As social restrictions on touch were loosened among DeafBlind people, new pathways in the deictic field became available, and these pathways affected the internal organization of the deictic system. I use the term “deictic integration” (Edwards, 2014b: pp. 27–61, 159–190), to account for the coordination of linguistic elements that derive from the deictic system with non-linguistic elements that derive from the deictic field into tighter and more restricted configurations over time so that (a) when a deictic sign is instantiated, retrievable values are restricted to a small and alternating set, and (b) deictic signs are organized by contrastive opposition (e.g., *this* and *that* in English). For example, the pronominal system of VASL makes a two-way distinction between first and non-first person (Meier, 1990: p. 377). The first person form is encoded in a pointing sign directed toward the signer and the non-first person form is encoded in a pointing sign directed away from the signer. This distinction retrieves values from basic participant frameworks, which inhere in the deictic field. In other words, these pointing signs are organized by contrastive opposition, which derive from the linguistic system, and are set to retrieve one of a restricted set of values (i.e., first or non-first person) from the deictic field. The participant frameworks themselves derive from the deictic field, and only the most schematized, basic, or expectable configurations make their way into the deictic system (Hanks, 1990: p. 149).

Linguistic pointing signs are therefore distinguished from pointing gestures according to the tightness of the relations that obtain between (1) schematic, oppositional categories, which are repeatable and transportable across contexts, and (2) relations, roles, and dynamics in the deictic field, where those forms are routinely instantiated. If a pointing sign is momentarily altered as it is brought into alignment with some dimension of context, linguistic and deictic elements are merely *coordinated*. If there is a restricted set of values (e.g., person and number values), and one of those values must be selected in order to produce a grammatical utterance, linguistic and deictic elements are *integrated*. The process whereby the deictic system and the deictic field are coordinated into tighter and more restricted configurations is what I am calling “deictic integration.”

Together, embedding in the social field and deictic integration narrow interactional and referential possibilities, thereby reducing the cognitive burden that interactants are faced with as they attribute intentional states to one another. This process became evident in the pro-tactile workshops as a range of linguistic forms were deemed inappropriate and fell out of use. It is not trivial that the first forms to go were VASL pointing signs. From the perspective of the DeafBlind language-user, many forms that derive from VASL feel intuitive despite the fact that they do not describe or articulate to a perceptible world. This is because the habitus is not redundant with, or even consistent with, sensory capacity. For example, it may feel natural or intuitive for DeafBlind people to sit down in the same manner that sighted people do. However, among exclusively DeafBlind people, this practice leads to collisions. This highlights the fact that the habitus can be in direct conflict with the capacities of the body and its ways of interacting with the physical world.

The same disconnect affects deictic reference. At the beginning of the pro-tactile workshops, many participants referred to objects in the immediate environment as if their interlocutors could see what they were pointing at. It took a person imbued with authority to change such practices by deeming them inappropriate and suggesting an alternative. From there, modes of access were brought into alignment and made reciprocal each time an object of reference was individuated by way of mutually accessible relations. This process, which involved the embedding of language in both the social and deictic fields, narrowed the range of potential linguistic resources to those that were “fieldable” (Bühler, 2001 [1934]) and it narrowed the range of retrievable contextual values to those that were mutually accessible (Hanks, 2005b). When a fieldable pointing sign is instantiated, the addressee is not abandoned in unstructured space with no clues for how to proceed; rather, they are the recipient of a signal, telling them to choose one path over another in a highly restricted field of possibilities (Bühler, 2001 [1934]).

## Appropriate pointing in TASL: embedding and integration in the social and deictic fields

**Figure 1:**
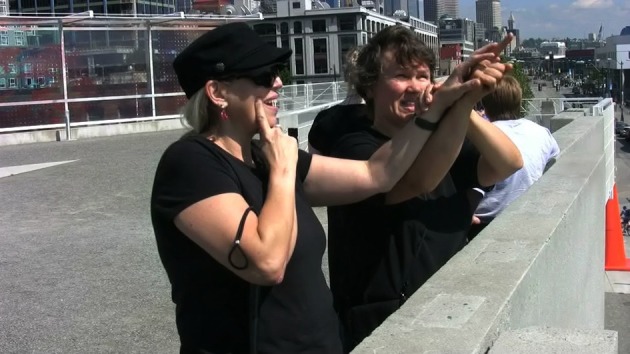
**VASL pointing sign**.

Prior to the pro-tactile movement, pointing signs were produced for DeafBlind people by sighted interpreters, as would be expected in Visual American Sign Language (VASL). For example, in Figure 1, a sighted interpreter (right) is pointing to a referent in the environment by extending her pointing finger toward it, along a visually accessible trajectory. The DeafBlind person (left) receives the sign tactually.

In the pro-tactile workshops, this type of pointing was deemed inappropriate by the instructors, Adrijana and Lee, and pro-tactile philosophy became a way of legitimizing alternate practices. For example, in the following exchange Adrijana demonstrates to her student that he can't resolve reference using VASL pointing signs and she explains that this failure is predictable from the perspective of pro-tactile (or “PT”) philosophy.

Adrijana: I'm going to explain PT philosophy to you. I'm not going to preach. It's going to be a discussion between the two of us. So let's say that I come up to you, and I start explaining: “There's a table over there [pointing], and there's a wall over there, and there's a door further over there.” Do you understand me?

DB Participant: Yes.

Adrijana: No you don't…

DB Participant: You said that there is a wall over there [points] and a door over there [points] right?

Adrijana: No, the door is over there [points].

DB Participant: Well, whatever.

Adrijana: Yeah, but that's exactly it. It's important. When people point like that to direct you, and you're standing in the middle of the room, you're totally lost. Right? [DB participant nods]. You're sitting here, and it might seem clear for a minute, but when you stand up and try to find the things I just located for you, the directions won't seem to match the environment and you'll be confused. Deaf [sighted] people do that—they point to places, but that's not clear.

DB Participant: Well, yeah. That's visual information.

**Figure 2:**
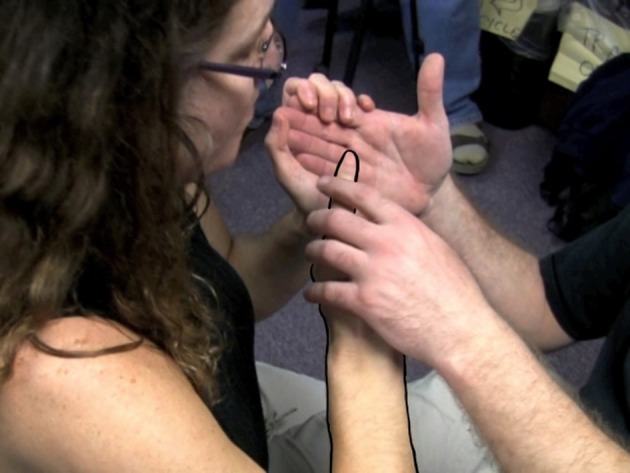
**TASL Pointing Sign**.

Adrijana: Right, but it has to be adapted to be pro-tactile. So instead of pointing, we have to teach them to do this (See Figure 2).

To demonstrate the appropriate procedure for referring to the location of the door, Adrijana substituted VASL pointing signs like the one in Figure 1 for TASL pointing signs like the one in Figure 2.

Notice that in the above exchange, Adrijana flat out contradicts the claim made by her student that he understands, and her student responds by adopting the practice she proposes. As discussed above, that move is successful is because Adrijana is invested with the requisite authority. This is a social fact, which has a particular history (see Edwards, 2014b: pp. 65–113). This exchange is part of a larger discourse that grew during the pro-tactile workshops, aimed at associating specific tactile communication practices with “pro-tactile people” so that using particular forms is not only a means of accomplishing reference by linking people, language, and the physical environment, but also a means of taking up new and increasingly valued social positions (Edwards, 2014b). In interactions like these, novel linguistic forms are *embedded in the social field*: to be a pro-tactile person is to point in a particular way. However, the designation of the form as pro-tactile also derives from the fact that it is fieldable, and is therefore a feasible candidate for a process of *deictic integration*. Novel, pro-tactile pointing signs articulate to the deictic field of TASL, as opposed to the deictic field of VASL. Where embedding in the social field and deictic integration come together, novel linguistic forms tend to emerge.

**Figure 3:**
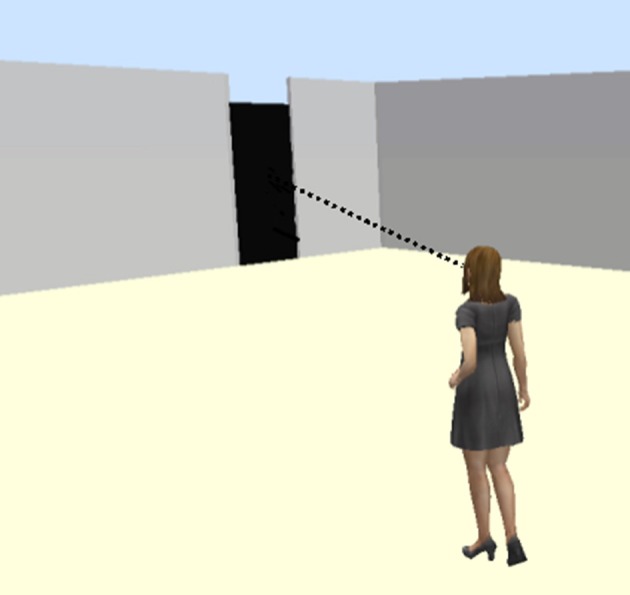
**Visual Pathway**.

The deictic field of VASL is organized around visual modes of access to the immediate environment. For example, in Figure 2, Adrijana points to a location on the addressee's palm and associates it with where they are at the time. She then locates the wall and the door relative to that against the tactually accessible backdrop of the addressee's hand. Then she says, “That's more clear, right? Better than [VASL] pointing?” And the participant says, “Yes. It helps because it's kind of like drawing a map. Then you can really visualize where things are.” Notice that the handshape in both the VASL and TASL pointing signs is roughly the same: one extended index finger directed toward the location of a referent. However, the trajectories launched by the handshape articulate to distinct pathways. Given the body-schema of a sighted person, the sightline in Figure 3 will feel like a commonsensical trajectory with which a pointing sign can align.

**Figure 4:**
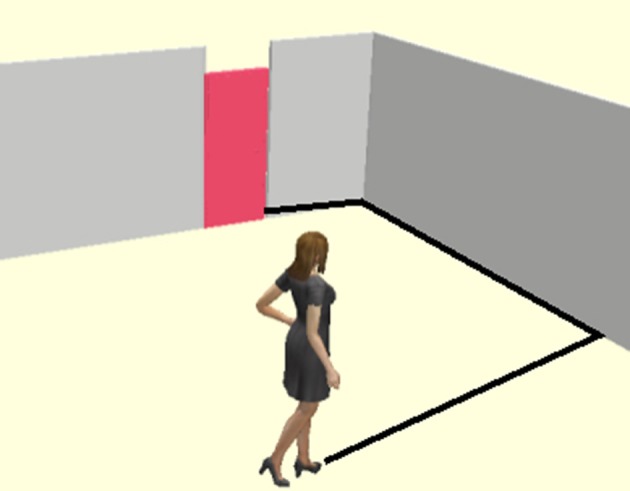
**Tactile Pathway**.

Given the body-schema of a pro-tactile DeafBlind person, however, the sightline in Figure 3 is likely to be inaccessible and/or inappropriate. Instead, some kind of tactually accessible pathway must be located, such as the one in Figure 4, which includes a straight orienting line that can be identified with a cane and tracked. Over time, patterns in how lines of travel intersect, where doors tend to be located, how materials are organized into common sequences, and so-on, become intuitive as they are incorporated into the habitus, and an orienting grid becomes available. In order for reference to be reliably resolvable, participants must be able to act *as if* orienting grids are reciprocal across the group of language-users, and this as-if clause has some minimal threshold of actuality built in. If everyone acts as if they are sighted when they are actually DeafBlind they will not be able to locate the door. Nevertheless, sensory capacities will not be consistent across the group—some will have more or less vision, better or worse vestibular function, and so-on. Therefore, a reciprocal orienting grid need not be identical, just calibrated to a coordinate scheme that is good enough for all involved. In other words, the body schema must be reciprocal, and it must be calibrated to the interactional and social fields inhabited by DeafBlind people.

**Figure 5:**
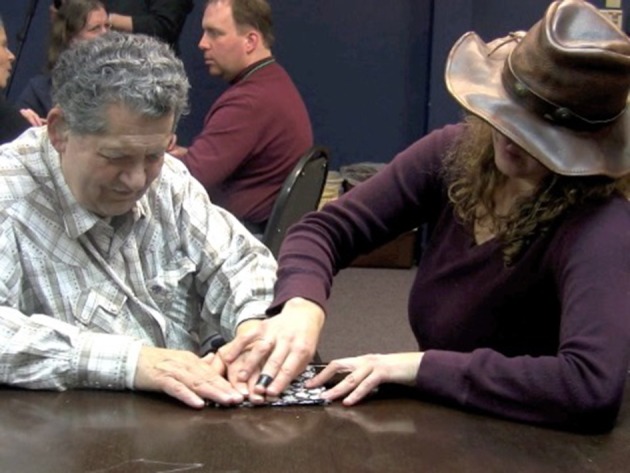
**Adrijana guides Hanks' hand across top edge of napkin**.

**Figure 6:**
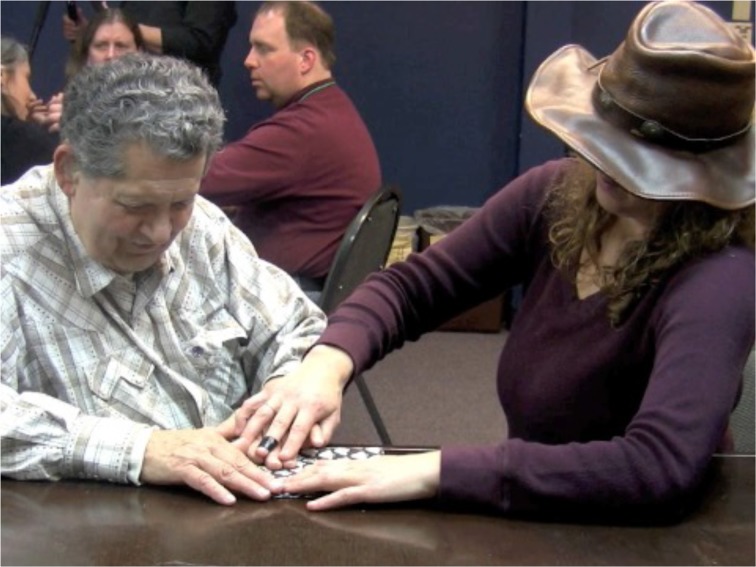
**Adrijana guides Hanks' hand across bottom edge of napkin**.

Prior to the pro-tactile workshops, DeafBlind people oriented to the environment in many different ways, which were more or less commensurate with their sensory capacities. Those who relied heavily on sighted people as guides were less likely to develop navigational habits organized around tactile modes of access, while those who relied less on sighted people were more likely. Therefore, body-schemas were not consistent across the group. This became apparent in many ways to participants of the pro-tactile workshops, and that recognition led to new practical routines. For example, before anyone started talking about or referring to an object, participants would often explore it tactually. In the following sequence, Adrijana leads a napkin-folding exercise, which involves learning how to do a “pocket fold.” In Figure 5, she grips the top of Hanks' hand, and guides it carefully along the top edge of the napkin. In Figure 6, she guides his hand along the parallel edge of the napkin. The two sides have different thicknesses because one side includes hemmed edges and the other one doesn't. She does the same thing with the remaining two sides of the napkin.

**Figure 7:**
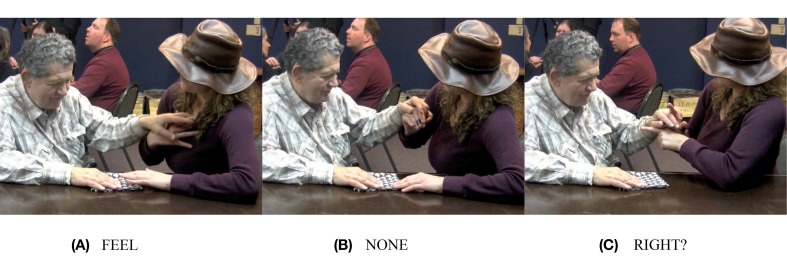
**Adriana asks Hank if he can feel the difference in thickness between the two edges**.

**Figure 8:**
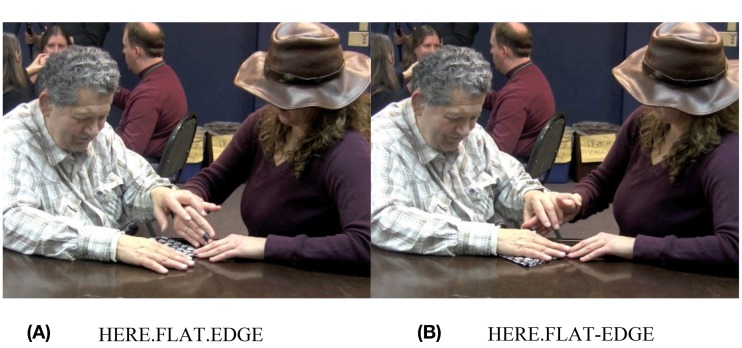
**Adrijana shows Hank where the flat edges are**.

**Figure 9:**
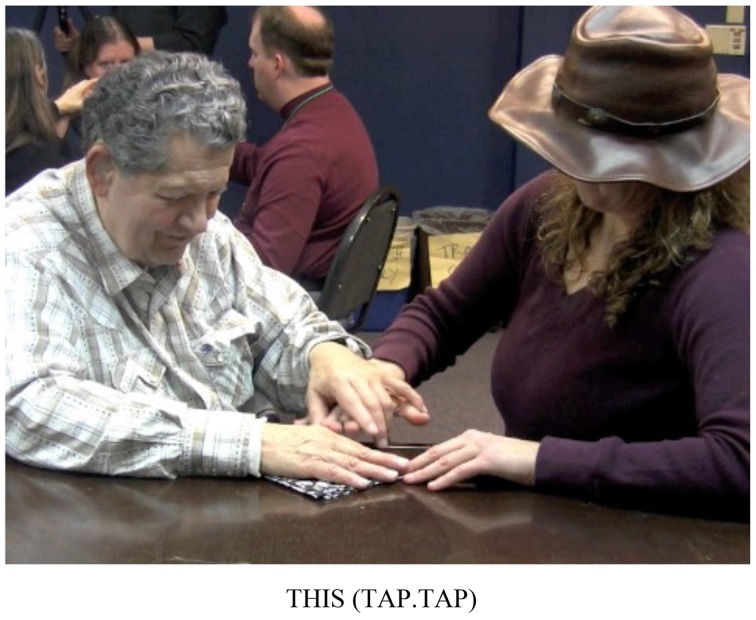
**Adrijana refers to a corner of the napkin by tapping on it twice with a flat hand**.

In Figure 7A, Adrijana signs FEEL, and in Figure 7B, she signs NONE. Then, in Figure 7C, she says, “RIGHT?” followed by a question marker (not pictured here), meaning, “You don't feel any [thickness] there, right?” Then she runs her fingers over the bottom edge and the left edge of the napkin, drawing attention to the fact that both of those sides are flat and smooth, unlike the hemmed edges. Hank acknowledges this, by signing YES (not pictured). Then, Adrijana rotates the napkin so that the two flatter edges extend away from Hank, and the corner is pointed toward the edge of the table. Hank's hands remain on top of Adrijana's as she rotates the napkin and also remains in contact with the table under it, so he can feel the relative position of the napkin shift. In Figure 8, she uses a flat handshape to refer to them by moving the edge of her hand back and forth in line with the edges, meaning something like, “Here is one flat edge and here is another flat edge.” In this sequence, Adrijana draws Hanks' attention to a tactually perceptible difference in two aspects of the object: two of the napkin's edges are thicker because they include hemmed edges, and two of the napkin's edges are flatter, because they do not include hemmed edges. Adrijana then taps twice on the corner of the napkin where the two flat edges come together (Figure 9), which I have glossed, THIS.

In contrast to many other attempts to single out a bounded referent, this attempt worked, evidenced by the fact that later in the interaction, Hank was able to perform the napkin fold successfully, and also by consistent signals of understanding throughout this stretch of the interaction. The reason for its success is that the field of potential referents was restricted significantly by interactional and social processes. When Adrijana signs THIS, she signals to Hank to choose one aspect of the object over another: this corner and not some other aspect of the object we have previously singled out. This restriction emerged over the course of several turns, prior to the moment in Figure 8. In addition, before this interaction, admissible dimensions of the object were restricted to those that could be accessed given a particular habitus, and the orienting scheme that DeafBlind participants were building over the course of the workshops. Pro-tactile people were beginning to narrow things down in ways that visual people wouldn't think to.

The deictic sign registers these restrictions in two senses: first, it is fieldable, i.e., it articulates to a field organized around tactile modes of access by being directed toward a location that both speaker and addressee can touch and distinguish from other aspects of the object. In contrast, a pointing sign that launches a trajectory into a visually organized space would not be fieldable. Second, the form of this deictic sign is perceptible and easily contrasted with other, perceptible forms. In this example, two taps on the referent functions as a demonstrative—Adrijana is trying to single out *this* part of the object. While more data is needed, this appears to be an emerging pattern. In contrast, tracing movements on the body of the addressee are used to identify the location of one referent in relation to another (for example, the door, relative to “us” in Figure 2). This suggests that tapping vs. tracing may be taking on a contrastive relation in TASL, which corresponds to demonstrative vs. locative functions^12^.

The ability of the addressee to attribute an intentional state to the signer is augmented by emergent distinctions like these in the language. It is also reinforced by an emergent, pro-tactile habitus and the fields with which it articulates. In order for Adrijana to be successful in teaching Hank to do a pocket-fold, he must be able to grasp the directedness of her mental states to answer questions like: *what is she focusing on and singling out for me?* A perceptible contrast between demonstrative and locative clues is invaluable when faced with such tasks. In addition, Hank does not have to entertain the possibility that Adrijana might direct his attention to dimensions of setting that she knows he can't perceive. This was not a safe assumption prior to the pro-tactile movement. These kinds of mutual alignments between the body, language, and the social world are helping participants rule out many logically, linguistically, and physically possible intentional states that could be attributed to their interlocutors.

## Conclusion

In examining social and deictic processes of embedding among DeafBlind people, I have shown how embodied forms of knowledge can simplify pragmatic acts of intention attribution, particularly with respect to deictic reference. I have argued that as social, interactional, and physical pressures are exerted on the language via the body, a process of integration is set in motion and the internal structure of the language is reconfigured. This suggests that language and context are not linked by way of external rules, maxims, or conditions. Rather, the linguistic system is continually adapted to, and shaped by, the historically specific fields of activity in which it is used. In other words, as contextual values are retrieved in interaction, patterns begin to sediment. From within those patterns, some values become more likely candidates for retrieval than others. In this sense, the language develops receptors, with particular sensitivities built in; a tactile language is not set to retrieve values from a field organized around visual modes of access.

In this article, I have argued that one of the key components of this process is deictic integration, or the coordination of linguistic and deictic elements into tighter and more restricted configurations over time. When an individual acquires a deictic system, they are acquiring a relational configuration of receptors, set to retrieve certain dimensions of context and not others. From this perspective, a range of pragmatic inferences will feel commonsensical, while others will feel like strange leaps that only philosophers would make. Following Bourdieu (1971, 1990 [1980]); Giddens (1979), and Hanks (1996, 2005a,b), I locate this commonsense, practical knowledge in the body, where it is registered neither as a representation, nor as a physical fact, but as a reflexive grasp that social actors have of being in a concrete world, which is often expressed as a dispositional tendency.

The cognitive tasks required for generating pragmatically situated meanings are attenuated when viewed from within the constraints of an individual's dispositional tendencies. This is particularly true if, as I have argued in this article, the social configuration of the body grounds relations between the language-user, the linguistic system, and the modes of access that are reciprocal across the group. Caught up in these complex relations, the body exerts an indirect but consequential effect on the contextual receptors that develop in any linguistic system; language anticipates context. I am not arguing, however, for an assumed or pre-determined fit between conceptual representations (linguistic or not) and the world^13^. Rather, the integration of language and context is the outcome of socio-historical and interactional processes, which from the perspective of the addressee, reduce the range of feasible, intentional objects (i.e., objects to which mental states are co-directed).

The approach sketched out in this article can also be distinguished from traditional approaches to speech acts. Searle's language-user, for example, would never come out of an interaction concluding that the reason their assertion or command was unsuccessful was that the linguistic system itself was inadequate to the task. Likewise, he would not presume that a description was unsuccessful because the world was not accessible in reciprocal ways. However, these are precisely the assumptions DeafBlind leaders acted on. The practices that were subsequently established linked language to context in new ways, and in the process, a range of potential interpretations and attributions were ruled out—not by a static and detachable set of conditions, rules, or maxims, but by the reconfiguration of the language as it was embedded in, and integrated with, new social and interactional fields.

### Conflict of interest statement

The author declares that the research was conducted in the absence of any commercial or financial relationships that could be construed as a potential conflict of interest.

## Footnotes

^1^See Duranti (2010) for a critique of intersubjectivity in the social sciences, which touches on this and related points.

^2^This study has been approved by the Committee for Protection of Human Subjects at the University of California, Berkeley, and all research subjects have given their informed consent.

^3^When these data were collected, I made an extensive list of transcribed entries, describing and translating interactions that were of interest, given my theoretical interests at the time. I also included things that seemed to diverge in significant ways from what would be expected in visual signed language communities. Each entry includes a video code and a time code, and the overall document functions like an index of notable interactional events. This index is 60 pages long and contains roughly 100 descriptions and/or translations of communicative sequences. For purposes of this article, I reviewed this index, and drew on it for some of the examples. In addition, I returned to the raw data, looking for patterns specific to demonstratives and locatives; please see footnote at the end of Appropriate Pointing in TASL: Embedding and Integration in the Social and Deictic Fields. I have also participated in the practices described here over the past 15 years of in-depth ethnographic engagement, and have developed an intuition for patterns in language that are new, and those that are not. I am relying on all of these forms of knowledge in my analyses.

^4^These are intentional states insofar as they are directed. Diffuse anxiety or elation, with no identifiable cause does not count as an intentional state for Searle.

^5^Cf. Austin's first Gamma condition (1965: p. 15) and Grice's maxims of quality (1989: pp. 27–28).

^6^Cf. Grice's cooperative principle (1989: p. 26).

^7^Dialog was translated from American Sign Language into English by the researcher.

^8^See Edwards (2014b: pp. 118–143) for further discussion.

^9^This exchange took place in the second of 10 pro-tactile workshops, when basic problems were being identified. The only sighted people present during the workshops were working as part of the research team, videorecording the interactions.

^10^See also Sauerburger (1993: pp. 87–98) on isolation in DeafBlind populations.

^11^See Edwards (2012) for discussion.

^12^The pro-tactile workshops were naturalistic interactional contexts where there were many variables in play (as opposed to an experimental context, where variables are more tightly controlled). Therefore, I am hesitant to make a definitive claim here, and am currently conducting more controlled elicitations to follow up on these findings. However, this provisional claim is based on what appears to be a fairly stable pattern in certain portions of the data. I discovered this pattern first by developing an intuition as I participated in these practices with DeafBlind people. I followed up on that intuition by jumping to places in the video footage where I thought demonstratives and locatives might appear, including the beginning of each workshop, where the instructors would give directions for the day, and activities that included instructions on how to manipulate objects, such as a crocheting exercise, and a direction-giving exercise. I looked for questions like “which one?,” “where?” or moments where it seemed that the signer was trying to single out one thing as opposed to something else—e.g., if there were two chairs, one sitting next to the other one, and a signer tried to draw attention to the one they wanted their interlocutor to sit it, I recorded the form that was used to accomplish that task. In the first 6 classes, I noticed that there were a lot of avoidance behaviors, even when asked specifically to provide locational information, or to single out a referent among others. There were also many cases where visual pointing signs were used and these forms were usually followed by confusion, requests for clarification, re-statement, or the use of English calques, that do not require explicit locational information to be disclosed. In the first 6 classes, I recorded 21 occasions on which signs were used to single out a thing among others, or to provide information about its location relative to other things, and I took note of the form that was employed. The “TAP.TAP” form that later took on a stable demonstrative meaning was only produced by one signer, three times in that data set. Then, in the 7th class alone, I identified 42 tokens of TAP.TAP produced by 7 signers, all of which occurred in contexts that suggested a demonstrative meaning. I identified 36 tokens of tracing, like the kind described in the napkin example, produced by 4 signers, in contexts that suggested locative meanings. In addition, when these forms were used, the addressee often produced backchanneling signals used for agreement, understanding, and continued attentional focus in response. However, these interactional contexts are not comparable across the different classes in the pro-tactile workshops, and there are too many variables to be sure about when, exactly the pattern emerged, and if, in fact, these forms map consistently onto a distinction between demonstrative and locative meanings. Therefore, further evidence is currently being collected and analyzed.

^13^As, for example, in Liddell's “real space” (bib31: p. 84).
